# Pharmacological inhibition and reversal of pancreatic acinar ductal metaplasia

**DOI:** 10.1038/s41420-022-01165-4

**Published:** 2022-09-02

**Authors:** Lais da Silva, Jinmai Jiang, Corey Perkins, Kalina Rosenova Atanasova, Julie K. Bray, Gamze Bulut, Ana Azevedo-Pouly, Martha Campbell-Thompson, Xiaozhi Yang, Hesamedin Hakimjavadi, Srikar Chamala, Ranjala Ratnayake, Raad Z. Gharaibeh, Chenglong Li, Hendrik Luesch, Thomas D. Schmittgen

**Affiliations:** 1 Department of Pharmaceutics, College of Pharmacy, University of Florida, Gainesville, FL USA; 2 Department of Medicinal Chemistry, College of Pharmacy, University of Florida, Gainesville, FL USA; 3 Center for Natural Products, Drug Discovery and Development, University of Florida, Gainesville, FL USA; 4 Department of Pathology, Immunology, and Laboratory Medicine, College of Medicine, University of Florida, Gainesville, FL USA; 5 Department of Surgery, University of Arkansas for Medical Sciences, University of Florida, Gainesville, FL USA; 6 Department of Medicine, University of Florida, Gainesville, FL USA; 7 Department of Molecular Genetics and Microbiology, University of Florida, Gainesville, FL USA

**Keywords:** Cell biology, Gastrointestinal diseases

## Abstract

Pancreatic acinar cells display a remarkable degree of plasticity and can dedifferentiate into ductal-like progenitor cells by a process known as acinar ductal metaplasia (ADM). ADM is believed to be one of the earliest precursor lesions toward the development of pancreatic ductal adenocarcinoma and maintaining the pancreatic acinar cell phenotype suppresses tumor formation. The effects of a novel pStat3 inhibitor (LLL12B) and the histone deacetylase (HDAC) inhibitor trichostatin A (TSA) were investigated using 3-D cultures from p48^Cre/+^ and p48^Cre/+^LSL-Kras^G12D/+^ (KC) mice. LLL12B and TSA inhibited ADM in both KC and p48^Cre/+^ mouse pancreatic organoids. Furthermore, treatment with LLL12B or TSA on dedifferentiated acini from p48^Cre/+^ and KC mice that had undergone ADM produced morphologic and gene expression changes that suggest a reversal of ADM. Validation experiments using qRT-PCR (p48^Cre/+^ and KC) and RNA sequencing (KC) of the LLL12B and TSA treated cultures showed that the ADM reversal was more robust for the TSA treatments. Pathway analysis showed that TSA inhibited Spink1 and PI3K/AKT signaling during ADM reversal. The ability of TSA to reverse ADM was also observed in primary human acinar cultures. We report that pStat3 and HDAC inhibition can attenuate ADM in vitro and reverse ADM in the context of wild-type Kras. Our findings suggest that pharmacological inhibition or reversal of pancreatic ADM represents a potential therapeutic strategy for blocking aberrant ductal reprogramming of acinar cells.

## Introduction

The adult pancreas is characterized by a high degree of plasticity. In response to injury or inflammation, the exocrine pancreas will lose mature, functional characteristics and re-establish embryonic features. Moreover, there is a shift from an acinar to a dedifferentiated, ductal-like phenotype, changes commonly referred to as acinar ductal metaplasia (ADM). ADM is a natural occurrence that follows periods of inflammation such as pancreatitis to temporarily reduce the damage from excessive digestive enzyme secretion in the pancreas. Characteristics of ADM include morphological changes as well as reduction in expression of acinar genes (e.g., carboxypeptidases, amylase) and an increase in expression of ductal and progenitor markers (e.g., KRT19, SOX9, respectively). The combination of sustained inflammation and mutated Kras in mouse acinar cells results in irreversible ADM-promoting precursor lesions and pancreatic ductal adenocarcinoma (PDAC) [1–3]. A current model for PDAC suggests that neoplastic transformation arises from both an acinar and ductal origin following enhanced Ras signaling (recently reviewed in [4])

The intent of this study was to investigate how small molecules affect ADM in vitro. We focused on two pathways, histone deacetylation (HDAC) and Jak/Stat3. Conditional mice carrying disrupted Yap/Taz crossed with mutated Kras led to persistent ADM lesions, PanIN formation and tumor progression; such a phenotype was not observed for mutated Kras in the absence of Yap and Taz [5]. This phenomenon occurred due to Yap/Taz interaction with the transcription factor Tead that upregulates the expression of many genes in the Jak/Stat3 pathway. Upregulation of the Jak/Stat3 pathway leads to increased inflammatory response and acinar cell susceptibility to undergo ADM. In a similar manner, IL22 promoted PDAC initiation through Stat3-mediated induction of ADM [3] and Klf5 disruption in mice produced an increase of Ndrg2, an inhibitor of Stat3 activation, reducing ADM and PanIN formation [2].

The HDAC Sirt1 regulates ADM by deacetylating Ptf1a and β-catenin [6]. The HDAC inhibitors trichostatin A (TSA) and panobinostat downregulate SIRT1 at the transcript level in HPDE pancreatic ductal epithelial cells [7]. Experiments using TGFα-induced acinar transformation demonstrated that the class I HDAC inhibitor MS-275 effectively inhibited pancreatic ADM [8]. Treatment of mouse acinar spheroids with nicotinamide, a Sirt1 inhibitor, attenuated ductal gene expression when the cells were plated on plastic culture dishes [9]. Moreover, re-appearance of amylase and reduction of Krt20 was apparent in the transdifferentiated cells treated with nicotinamide.

We applied a 3-D in vitro ADM model by which primary mouse pancreatic acini dedifferentiate when cultured on the extracellular matrix Matrigel [1]. We show that a pStat3 inhibitor LLL12B [10, 11] and a HDAC inhibitor TSA impede ADM in p48^Cre/+^ and p48^Cre/+^;LSL-Kras^G12D/+^ (KC) mouse organoids. Furthermore, addition of the compounds to mouse or human tissue cultures after ADM had occurred, resulted in cellular and gene expression changes that suggest a reversal of the dedifferentiated phenotype to one that is more acinar-like.

## Results

### Inhibition of pancreatic ADM by LLL12B

**Fig. 1:**
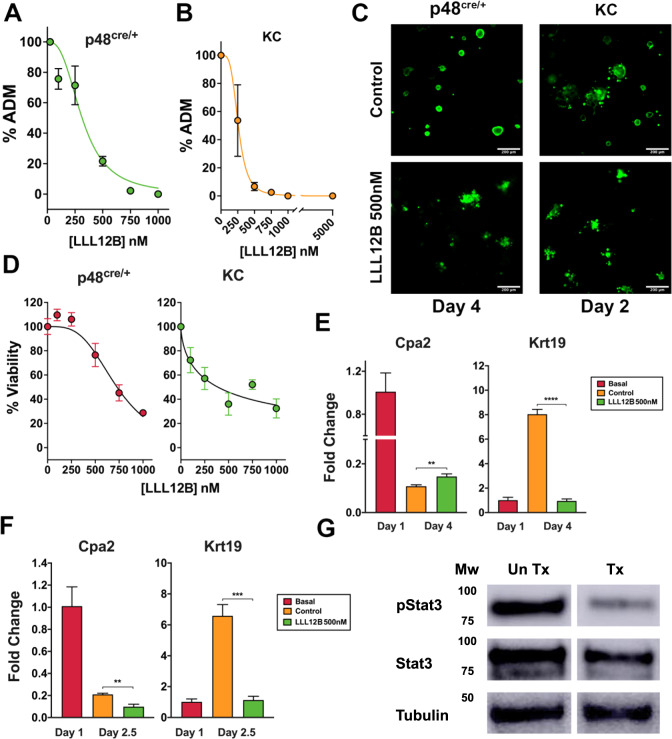
The pStat3 inhibitor LLL12B attenuates ADM. **A** p48^Cre/+^ mouse acinar cells were treated with LLL12B or untreated control for 4 days and the amount of ADM was microscopically quantified. **B** KC mouse pancreatic acini were treated with LLL12B or untreated control for 2.5 days and the amount of ADM was calculated microscopically. The viability of p48^Cre/+^ and KC mice treated with 500 nM LLL12B or untreated control for 4 and 2.5 days, respectively, was determined by **C** calcein AM staining or **D** MTT assay. The effects of LLL12B on the mRNA expression of Cpa2 and Krt19 in **E** p48^Cre/+^ (4-day exposure of 500 nM) or **F** KC (2.5-day exposure of 500 nM) mouse organoids as determined by qRT-PCR. The control group represents untreated control while basal represents the untreated day one cultures. **G** Western blot of KC mouse organoids from untreated (UnTx) or 1000 nM LLL12B treatment (Tx) for 6 h. The phosphorylation site for pStat3 is Tyr705. Mean ± SD from triplicate treatments. **P < 0.01, ***P < 0.005. ****P < 0.001.

The effects of pStat3 inhibition on in vitro ADM were studied using LLL12B treatment. Pancreatic organoids derived from 6-week-old p48^Cre/+^ or KC mice produced dedifferentiated ductal-like cells when cultured on Matrigel with ADM occurring at a faster rate in KC compared to p48^Cre/+^ mouse pancreata (SFig. 2). By day 2.5 of culture, about 50% of the acini from KC dedifferentiated (SFig. 2). To determine if LLL12B inhibits pancreatic ADM, the pStat3 inhibitor was added to the organoids from p48^Cre/+^ and KC mice. Treating the KC mouse organoids with LLL12B produced a comparable EC_50_ as the p48^Cre/+^ mice (p48^Cre/+^ EC_50_ = 314 nM, KC EC_50_ = 259 nM) (Fig. 1A, B); however, the treatments for the p48^Cre/+^ mice were over a 4-day exposure compared to 2.5 days for KC mouse organoids. Acinar cell viability, assessed using calcein AM staining, showed that viable clusters were maintained in the presence of 500 nM LLL12B treatment in both mouse organoids (Fig. 1C). MTT assay showed viability was reduced by 50% for concentrations of LLL12B greater than 500 nM for both the p48^Cre/+^ and KC treatments (Fig. 1D).

Next, we evaluated the mRNA expression of acinar and ductal genes in the LLL12B treated and control cells. Treatment with 500 nM LLL12B reduced ductal Krt19 expression in both p48^Cre/+^ (Fig. 1E) and KC organoids (Fig. 1F) while the effects on acinar gene expression was less consistent. LLL12B produced a slight but statistically significant increase in Cpa2 in p48^Cre/+^ (Fig. 1E) but reduced Cpa2 expression in the KC organoids (Fig. 1F). Verification of pStat3 inhibition by LLL12B was revealed by Western blotting (Fig. 1G, SFig. 1). In conclusion, LLL12B inhibits pancreatic ADM in both KC and p48^Cre/+^ mice which is accompanied by a reduction of pStat3 protein and ductal gene expression.

### Inhibition of pancreatic acinar transdifferentiation by TSA

**Fig. 2:**
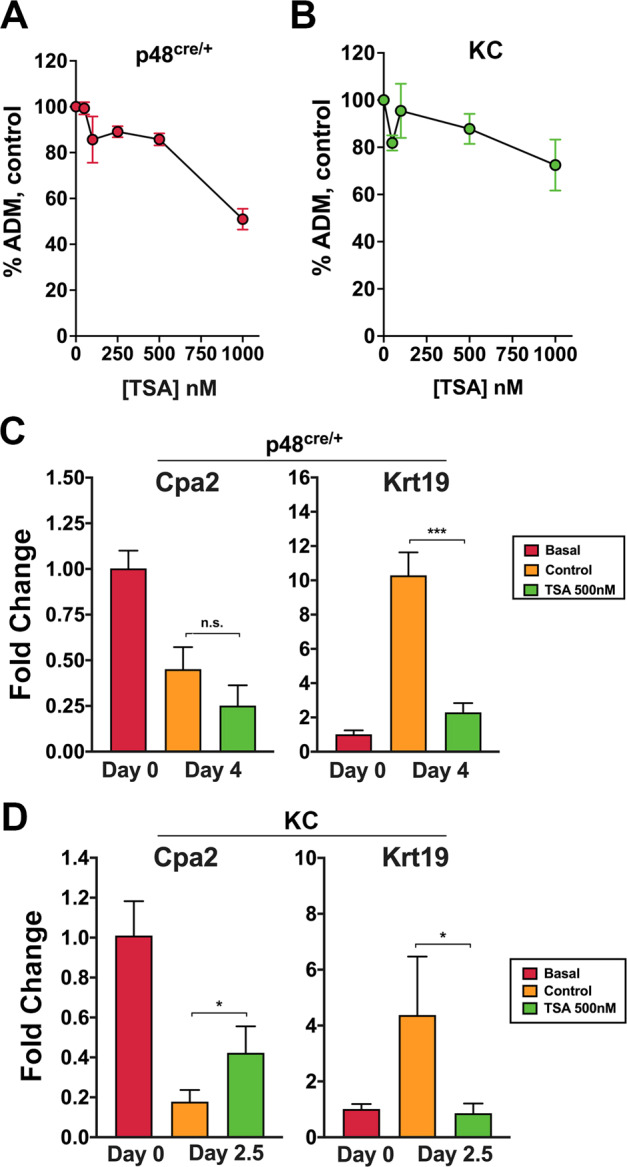
The HDAC inhibitor TSA attenuates ADM. p48^Cre/+^ (**A**) and KC (**B**) primary mouse pancreatic acini were treated with TSA for 3 and 2 days, respectively, and the amount of ADM was calculated microscopically. p48^Cre/+^ (**C**) and KC (**D**) acinar cultures were exposed to 500 nM TSA or untreated control for 3 and 2 days, respectively, and the expression of Cpa2 and Krt19 was determined using qRT-PCR. Mean ± SD from triplicate treatments. *P < 0.05; ***P < 0.001, n.s. not significant.

As increased HDAC activity has been reported during pancreatic development and differentiation [8], we evaluated the ability of the pan-HDAC inhibitor TSA to attenuate ADM. p48^Cre/+^ and KC mouse organoids were exposed to TSA under similar conditions as LLL12B. Treatment with 1 µM TSA reduced ADM by 50% in the p48^Cre/+^ organoids (Fig. 2A) and by about 30% in the KC organoids (Fig. 2B). Five hundred nM TSA reduced Krt19 expression in both mouse organoids studied (Fig. 2C, D). The effects on acinar gene expression were mixed with the Cpa2 expression not significantly changing in p48^Cre/+^ organoids yet TSA produced about a 2-fold increase in acinar gene expression in the KC organoids (Fig. 2C, D).

### Reversal of ADM by LLL12B in p48^Cre/+^ and KC organoids

**Fig. 3:**
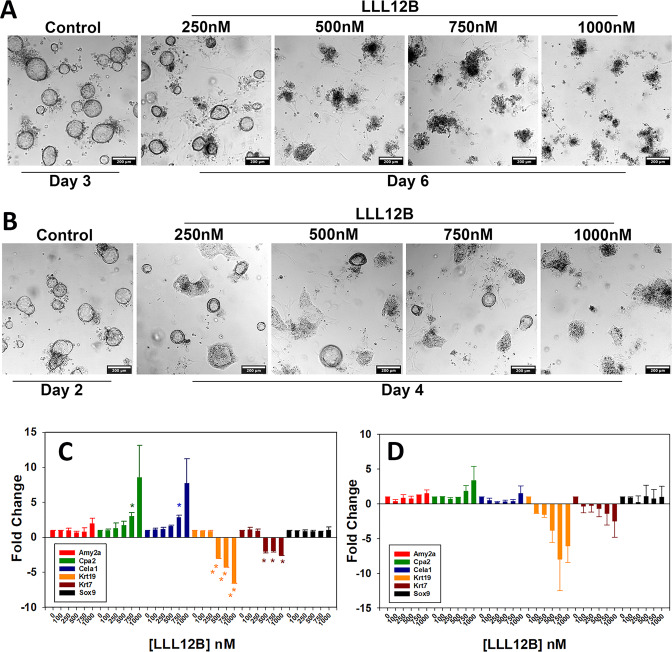
Reversal of ADM by LLL12B in p48^Cre/+^ mouse acini. **A** p48^Cre/+^ mouse pancreatic acini underwent ADM over 3 days of culture and were then exposed to increasing concentrations of LLL12B for 3 additional days. **B** KC mouse pancreatic acini underwent ADM over 2 days of culture and were then exposed to increasing concentrations of LLL12B for 2 additional days. Expression of acinar and ductal genes from LLL12B treated p48^Cre/+^ (**C**) or KC (**D**) mouse pancreatic organoids following the identical treatment as in (**A**) and (**B**), respectively. Data are presented as fold-change normalized to 18S rRNA and relative to the untreated control. Mean ± SD from duplicate experiments. *P < 0.05; **P < 0.01.

We next asked if pStat3 inhibition could reverse ADM after it had occurred. p48^Cre/+^ acinar organoids that underwent ADM for 3 days were treated with LLL12B for 3 additional days. On the day the cultures were exposed to LLL12B, the organoids had clearly transdifferentiated into ducts (Fig. 3A). However, after 3 days of LLL12B treatment, concentrations of 500 nM and greater produced less visible ducts and notably more opaque cellular clusters (Fig. 3A). LLL12B treatment of KC cultures did not produce the similar morphology as in p48^Cre/+^ and resulted in more cystic-like structures (Fig. 3B). We next used qPCR to investigate the acinar and ductal gene expression from the LLL12B treatments. In p48^Cre/+^ cultures, LLL12B produced a concentration-dependent increase in acinar genes (Amy2a, Cpa2, and Cela1), a reduction in ductal genes (Krt19 and Krt7) and no change in Sox9 expression (Fig. 3C). Treatment of KC organoids with LLL12B after ADM had occurred did not produce the same concentration-dependent increase in acinar gene expression as in the p48^Cre/+^ cultures, however, similar to p48^Cre/+^, LLL12B reduced Krt19 and Krt7 mRNA (Fig. 3D). We conclude that pStat3 inhibition following ADM can reverse the ductal phenotype to one that is more acinar in p48^Cre/+^ but not KC mouse cultures.

### TSA treatment reverses ADM in p48^Cre/+^ and KC organoids

**Fig. 4:**
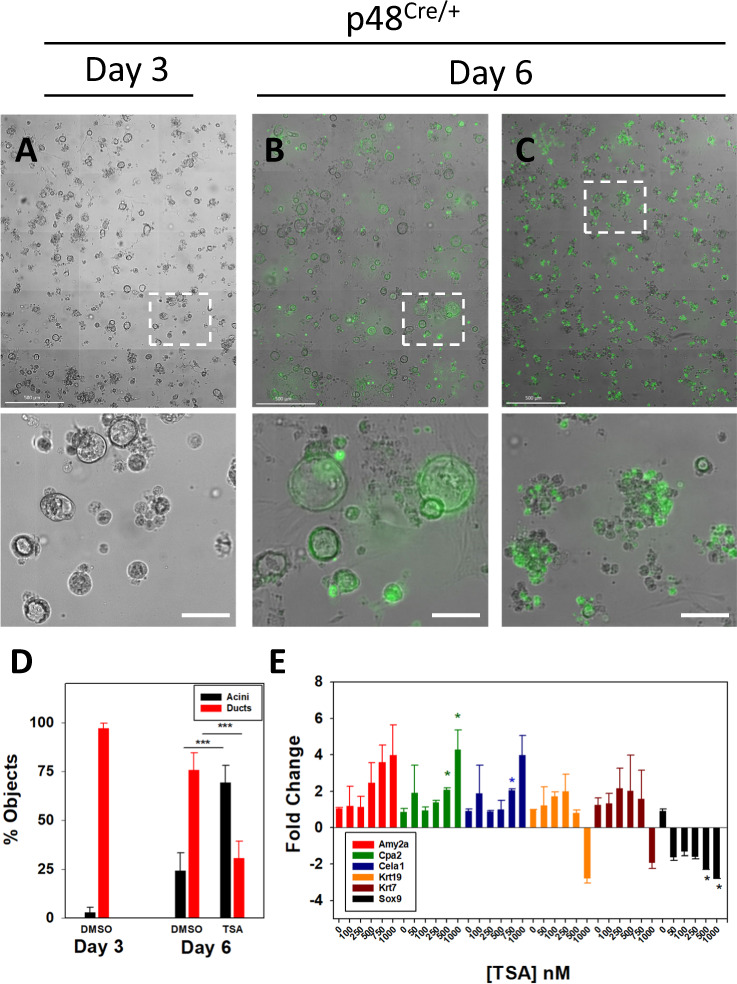
Reversal of ADM by TSA in p48^Cre/+^ mouse acini. p48^Cre/+^ mouse pancreatic acini underwent ADM over 3 days of culture (**A**) and were then exposed to DMSO (**B**) or 1 µM TSA (**C**) for 3 additional days followed by Calcein AM viability staining. Shown are the high content images, with the enlarged sections (dashed lines) in the lower panels. Scale bars in the top images represent 500 µm and in the lower images 100 µm. **D** Acini and ducts were microscopically counted from four sections of the high content images. **E** Expression of acinar and ductal genes from TSA treated p48^Cre/+^ mouse pancreatic organoids following the identical treatment as in (**A**–**C**). Data are presented as fold-change normalized to 18S rRNA and relative to the untreated control. Mean ± SD from duplicate experiments. *P < 0.05; ***P < 0.001.

**Fig. 5:**
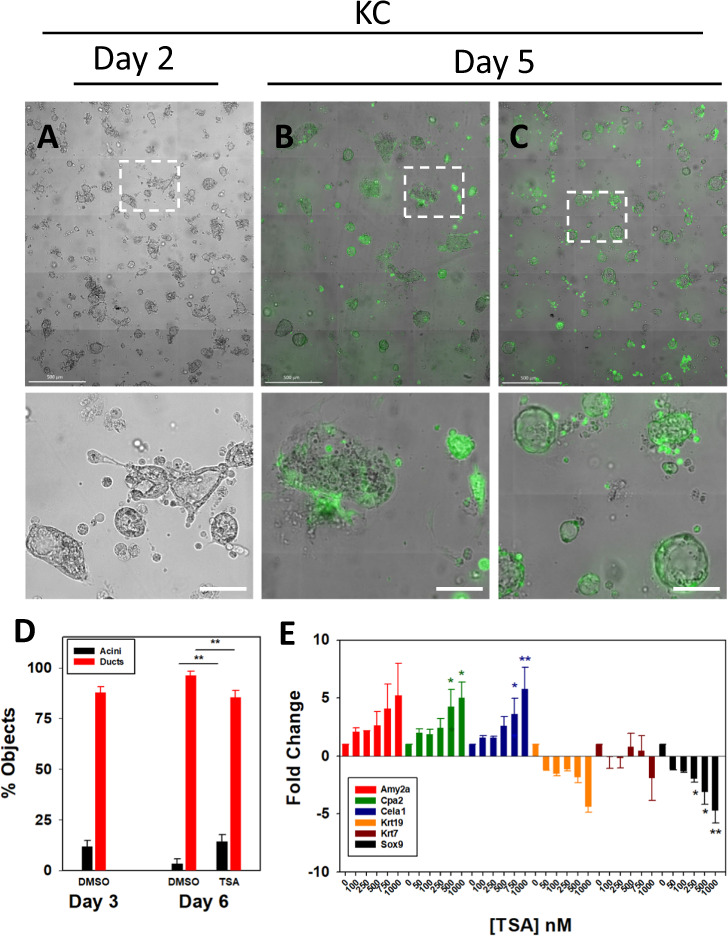
Reversal of ADM by TSA in KC mouse acini. KC mouse pancreatic acini underwent ADM over 2 days of culture (**A**) and were then exposed to DMSO (**B**) or 1 µM TSA (**C**) for 2 additional days followed by Calcein AM viability staining. Shown are the high content images, with the enlarged sections (dashed lines) in the lower panels. Scale bars in the top images represent 500 µm and in the lower images 100 µm. **D** Acini and ducts were microscopically counted from four sections of the high content images. **E** Expression of acinar and ductal genes from TSA treated KC mouse pancreatic organoids following the identical treatment as in (**A**–**C**). Data are presented as fold-change normalized to 18S rRNA and relative to the untreated control. Mean ± SD from duplicate experiments. *P < 0.05; **P < 0.01.

Next, the ADM reversal experiment was attempted on p48^Cre/+^ and KC organoids using the HDAC inhibitor TSA. The organoids were treated with TSA after when duct formation reached >50%; 3 days of culture (p48^Cre/+^) or 2 days (KC). Treating the p48^Cre/+^ mouse cultures with 1 µM TSA 3 days after ADM had occurred maintained cell viability as assessed by calcein AM staining and reversed the ductal morphology to one that was more acinar in appearance (Fig. 4A–C). Microscopic counting of acini and ducts following TSA treatments showed a reduction in ducts and increase in acinar cells (Fig. 4D). Likewise, the expression of acinar genes increased in a concentration-dependent fashion, however, only 1 µM TSA reduced Krt19 and Krt7 levels. TSA reduced Sox9 expression in a concentration-dependent fashion (Fig. 4E). Compared to p48^Cre/+^, treating KC mice with 1 µM TSA produced less clearly defined changes in cell morphology (compare Figs. 4C and 5C). Treating the KC cultures with 1 µM TSA after ADM had occurred produced more rounded ducts compared to DMSO control, but did not form the acinar clusters as in p48^Cre/+^ mouse cultures (Fig. 5B, C). Treatment of KC acini with TSA following completion of ADM increased acinar cell number, reduced ductal cell number (Fig. 5D), and increased acinar gene expression while reducing Krt19 and Sox9 expression (Fig. 5E). Treating the KC organoids with increased concentration of TSA (10 µM) resulted in acinar cluster morphology similar to p48^Cre/+^ ADM reversal (SFig. 3A–C), increased Amy2a (SFig. 3D) and reduced Krt7 (SFig. 3E) expression.

**Fig. 6:**
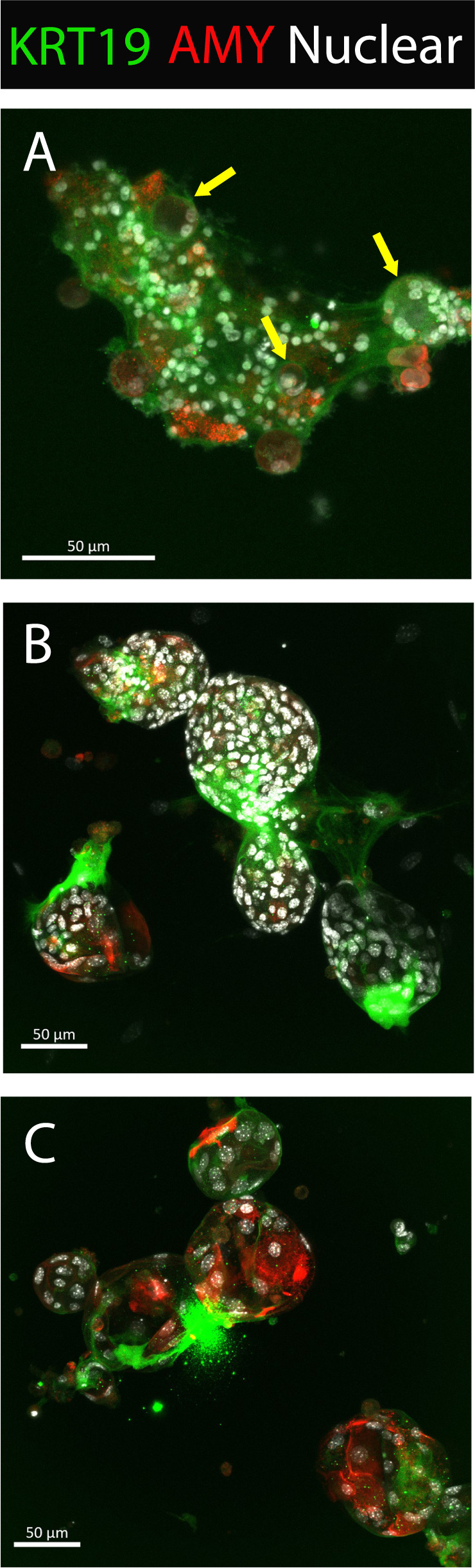
Acinar and ductal changes during dedifferentiation of KC mouse acini by TSA. KC mouse pancreatic acini were plated onto Matrigel and were fixed following one day of culture (**A**). Yellow arrows, ducts formed following ADM. After 2 complete days of culture the cells were either untreated (**B**) or exposed to 500 nM TSA (**C**) and cultured for 2 additional days before fixation and immunostaining. Scale bars: 50 µm.

Confocal imaging of the KC organoids was performed to determine if the protein expression correlated with the mRNA levels during the ADM reversal. Figure 6A shows acinar cells undergoing transition to early ducts evident by small spherical outgrowths from cells arranged in loose mass. Figure 6B shows multiple spherical structures with orderly nuclei in increased numbers. There appears to be aggregation of Krt+ positivity most likely where duct cells are congregated and with well-formed intercellular junctions. Figure 6C shows the loss of densely packed nuclei around a spherical structure as seen in Fig. 6B with increased Amy indicative of ADM reversal. Refer to SFig. 4 for individual channels for this image. We conclude that TSA reverses ADM in both p48^Cre/+^ and KC mice, however, the morphological changes in KC acini are more evident at the 10 µM treatment.

### TSA reverses ADM in dedifferentiated human acinar cells

**Fig. 7:**
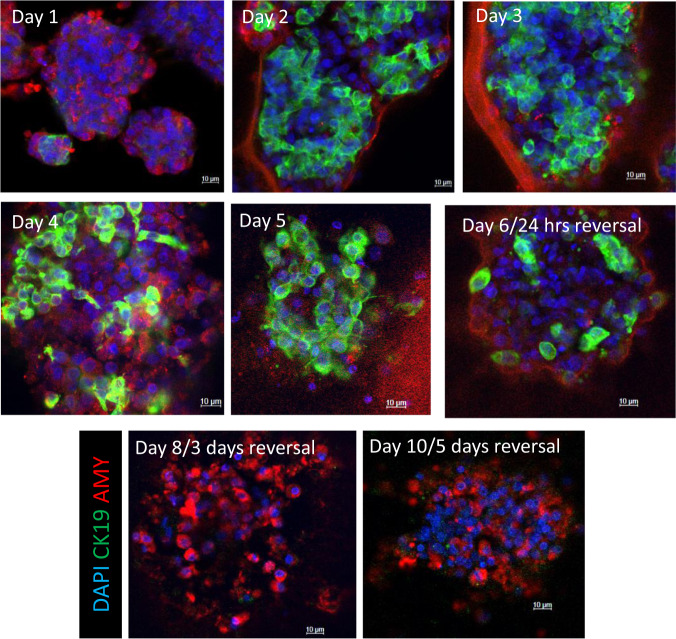
Reversal of ADM by TSA in primary human acinar cultures. Primary, human acini were cultured in Matrigel on glass chamber slides for 5 days. On day 5 of culture, 500 nM of TSA was added and the cells were cultured for 5 additional days. The cells were processed for immunohistochemistry and imaged for amylase (red) and KRT19 (green) using confocal microscopy.

Primary human islet depleted acinar cells were cultured on Matrigel to undergo ADM. Morphological changes show complete dedifferentiation into ductal-like cells over 5 days of culture (Fig. 7). On day 5, 500 nM TSA was added and the cells were cultured for 5 more days. After 24 h of drug treatment, a dramatic reduction in KRT19 staining was present. Remarkably, positive amylase staining with very little KRT19 protein was apparent after 3 and 5 days of treatment (Fig. 7).

### RNA sequencing confirms that TSA reverts KC cells to an acinar state and promotes acinar-related pathways

**Fig. 8:**
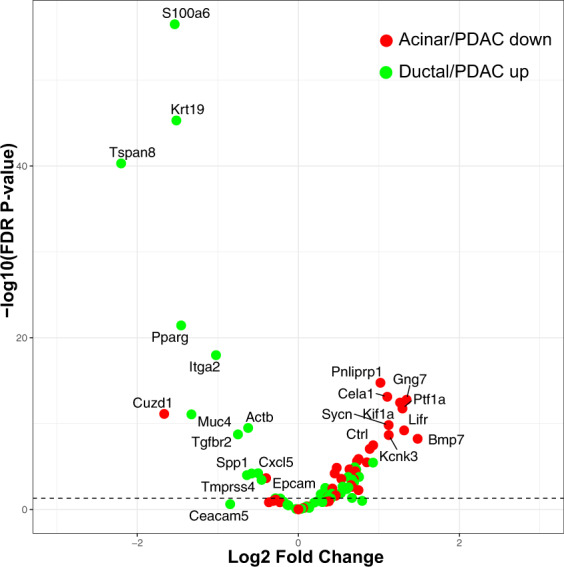
Volcano plot of differentially expressed genes from TSA-induced ADM reversal in KC mouse acini. KC mouse pancreatic acini underwent ADM over 2 days of culture and were then exposed to 500 nM of TSA for 2 additional days. RNA isolated from the treated and day 6 untreated control were subjected to Illumina NovaSeq6000 whole transcriptome sequencing. Shown are the expression of a selected set of 82 genes which are the mouse equivalents to those previously identified as associated with the pancreatic acinar/ductal phenotype or genes associated with the onset or progression of PDAC as described in the “Methods” section.

Whole transcriptome sequencing was performed on KC cells that were treated for 3 days with LLL12B or TSA following 3 days of ADM transformation in vitro. Quality control showed separate clustering of the samples with respect to treatment as shown in the PCA plot (SFig. 5). TSA treatment produced 4890 and 4052 upregulated and downregulated genes, respectively, while LLL12B resulted in 1698 and 1483 upregulated and downregulated genes, respectively. Volcano plots were used to examine the expression pattern of a selected set of 82 genes which are the mouse equivalents to those previously identified as associated with the pancreatic acinar/ductal phenotype [12, 13] or genes associated with the onset or progression of PDAC [12, 14, 15] (Supplemental Table 2). The data show a distinction between TSA-induced upregulation of acinar genes and those genes that are downregulated in PDAC while TSA treatment repressed the ductal and genes that are upregulated in PDAC (Fig. 8). Conversely, many of the acinar genes upregulated by TSA were downregulated by LLL12B (Amy2a, Pnlirp1, SFig. 6). These data further demonstrate that TSA treatment restores acinar gene expression following dedifferentiation to ductal-like cells.

**Fig. 9:**
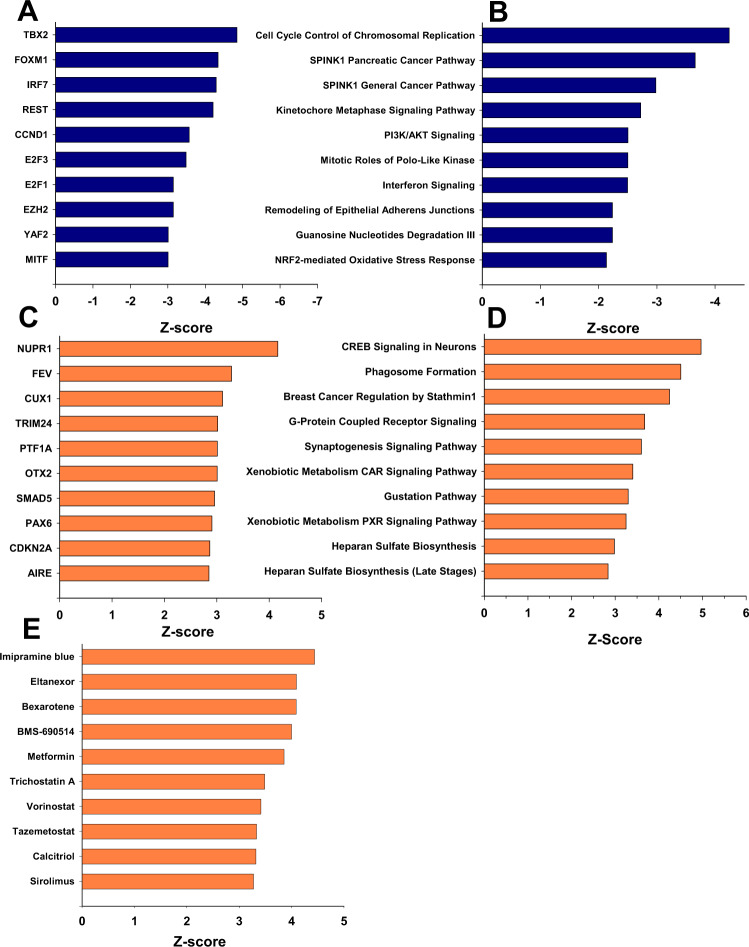
Pathway and upstream analysis during the reversal of ADM by TSA in KC acinar cells. RNA sequencing data from the ADM reversal in KC mouse acini following 500 nM TSA treatment was analyzed using Ingenuity Pathway Analysis. **A**, **C** Top 10 transcription factors involved in the upstream regulation ranked by Z-score. **B**, **D** Most highly ranked signaling pathways ranked by Z-score. **E** Top 10 chemical drugs predicted to regulate the signaling pathways. Blue, inhibited and orange, activated pathways.

We next used Ingenuity Pathway Analysis to determine if any ADM or pancreatic cancer pathways or transcriptional regulators were associated with reversal of ADM by TSA or LLL12B. Expression of transcription factors that promote the ductal phenotype (Rest [16]) or PDAC (Foxm1 [17]) were inhibited by TSA while the activity of the upstream transcriptional regulator Ptf1a that maintains the acinar phenotype was increased (Fig. 9A, C). Canonical pathways that were inhibited with TSA include cell cycle/division pathways such as cell cycle control of chromosomal replication and kinetochore metaphase signaling pathway as well as ADM-promoting pathways such as PI3K/AKT [18] and those associated with pancreatic cancer (e.g., Spink1) (Fig. 9B). Among the top 10 canonical pathways activated by TSA, none are associated with the acinar phenotype to our knowledge (Fig. 9D). The top ten upstream chemical drugs were ranked and TSA was the sixth most highly ranked chemical (Fig. 9E). Pathways affected by LLL12B were less ADM and pancreas-related compared to those affected by TSA (SFig. 7A–D). Stat3 ranked eighth in terms of inhibited upstream transcriptional regulators by LLL12B (SFig. 7C). A complete list of all pathways, transcriptional regulators, and drugs affected by TSA or LLL12B may be found in Supplemental Table 3.

## Discussion

ADM is microscopically observable in mice throughout experimental pancreatitis [2] and during the early stages of pancreatic cancer development in mice [1, 19–21]. ADM is also apparent in patients with PanINs [22], atypical flat lesions of the pancreas [23], and chronic pancreatitis [24]. ADM can be investigated in vitro using pancreatic mouse [1, 25–27,] or human [28, 29] organoids cultured on extracellular matrix, collagen/TGFα or in 3-D suspension [28, 30]. An in vitro pancreatic organoid ADM model was applied here to determine if pStat3 or HDAC inhibition could attenuate or reverse ADM. pStat3 and HDAC inhibitors were selected as these pathways have been implicated in mouse models of ADM, pancreatitis, and pancreatic cancer [5, 31–35].

Similar to other reports [2, 3, 5, 6, 8, 9], we found that both pStat3 and HDAC inhibition can impede ADM in vitro. The compounds inhibited ADM in both mouse strains studied. LLL12B (Fig. 1A, B) showed a concentration-dependent reduction in dedifferentiated structures in both p48^Cre/+^ and KC organoids. Compound treatment maintained cell viability using calcein AM staining, however, inhibition in ADM correlated with reduced viability at the higher drug concentrations by MTT assay (Fig. 1D). Thus, we cannot completely rule out the possibility that inhibition of duct formation resulted from pStat3 or HDAC inhibition alone but rather in conjunction with some toxicity. Bombardo et al. reported inhibition of ADM with the HDAC inhibitor MS-275 without a pronounced reduction in viability as assessed using MTT [8].

Our most novel finding is the compound-induced, morphological, and gene expression changes that demonstrate a reversal of ADM. To our knowledge, this is the initial report of pharmacological reversal of ADM in human pancreatic acinar cells and mouse acini containing either wild-type or mutant Kras. The overall effects of LLL12B and TSA on cell morphology and gene expression during ADM reversal were both compound, concentration and mouse-dependent. Based on the cell morphology and gene expression changes, we conclude that pStat3 inhibition reverses ADM in the p48^Cre/+^ but not the KC organoids (Fig. 3). These findings suggest a possible mechanism involving Stat3 signaling in physiological pancreatic regeneration and in vivo experiments using caerulein-induced pancreatitis are warranted. TSA treatment produced more pronounced ADM reversal in p48^Cre/+^, KC, and the human organoids. The RNA sequencing analysis also supported ADM reversal in KC by TSA but not LLL12B (Fig. 8, SFig. 6). TSA is a pan-HDAC inhibitor that regulates gene expression by maintaining acetylation marks on histones. Performing Chip Seq on activation (H3K4me3) and deactivation (H3K27me3) histone modifications showed that ADM is tightly regulated by histone acetylation [12]. Therefore, TSA and perhaps other HDAC inhibitors reverse the dedifferentiated phenotype of acinar cells during ADM by maintaining chromatin accessibility.

Notably, TSA reversal robustly increased acinar and reduced Sox9 gene expression in KC mice (Fig. 5E). Morphologically, 1 µM TSA altered ductal shape and size (Fig. 5B, C), while 10 µM TSA produced morphology reminiscent of acinar clusters (SFig. 3C). Our findings are significant as KRAS is mutated in more than 90% of PDAC and ADM is believed to be irreversible in acinar cells with mutant Kras. Moreover, acinar gene expression is largely absent in pancreatic tumors and preserving acinar cell identity has been shown to protect acini from oncogenic Kras-induced tumorigenesis [36]. Pathway analysis of acinar cells with mutant Kras showed that TSA inhibited more ductal and PDAC-associated pathways including PI3K/AKT signaling and Spink1 (Fig. 9). Our findings are reminiscent to those of Kim et al. [37, 38] who used the bHLH transcription factor E47 to re-express digestive enzymes in pancreatic cancer cell lines causing activation of the acinar maturation network and inhibition of tumorigenesis. Recently, Venis et al. developed a lab-on-a-chip approach to reverse a pancreatic cancer cell line to an acinar phenotype following re-expression of *PTF1A* [39].

Our study highlights the relevancy of in vitro models of exocrine cell plasticity. Aberrant pancreatic differentiation from acinar to ductal-like cells is believed to be an initiating event toward PDAC development [40–45]. Re-establishing control of cellular transdifferentiation could open the doors to induce malignant pancreatic ductal cells to a more benign cell state. Therapeutic inhibition or reversal of pancreatic ADM may represent a strategy for attenuating ductal reprogramming of acinar cells to prevent the initiation of PDAC in high-risk patients such as those with hereditary syndrome or chronic pancreatitis.

## Materials and methods

### Mice

p48^Cre/+^ mice were bred to LSL-Kras^G12D/+^ to produce p48^Cre/+^; LSL-Kras^G12D/+^ mice (KC). Genotyping for the presence of the transgene was performed by Transnetyx (Cordova, TN). Transgenic animals were bred and studies were performed at the University of Florida according to an approved IACUC protocol 201809058.

### Mouse acinar ductal metaplasia culture

Acinar cells were isolated from the pancreas of 6–8-week-old p48^Cre/+^ or KC mice using modifications of published protocols [25, 26]. After anesthesia with isoflurane and cervical dislocation, the whole mouse pancreas was collected, washed three times in cold Hanks Balanced salt solution (HBSS), and minced in a glass petri dish on ice. The minced tissue was aspirated using a 10 mL glass pipette, transferred to a 50 mL centrifuge tube containing cold HBSS, and then centrifuged at 720 × *g* for 2 min at 4 °C. Next, the tissue was dissociated for 30 min using 0.2 mg/mL collagenase P (Roche) in HBSS at 37 °C for 30 min. Mechanical dissociation was continued by mixing the contents using different-sized glass pipettes (10 mL wide bore and then 10 mL and 5 mL narrow bore). The dissociated tissue was washed 3 times in media containing 5% fetal bovine serum (FBS) to inactivate collagenase and was sequentially passed through 500, 300, and 200 µm mesh sieves (pluriSelect, Leipzig, Germany). The cells were embedded in Growth Factor Reduced Matrigel (Corning), and seeded into a 48- or 96-well plates. Modified Waymouth’s media (dexamethasone 20 µg/mL, 10% FBS, 0.1 mg/ml Soybean Trypsin Inhibitor (Sigma), penicillin 10,000 U/mL, and streptomycin 10,000 µg/mL) was added on top of the Matrigel and replenished every other day. For the experiments with 48-well plates, 200 µl Matrigel and 500 µl modified Waymouth’s media was used and for experiments with 96-well plates, 100 µl Matrigel and 100 µl modified Waymouth’s media was used.

### Human acinar ductal metaplasia culture

Primary human islet depleted pancreatic acinar cells obtained from organ donors were supplied by Prodo Laboratories (Aliso Viejo, CA) on a protocol (IRB201802669) approved by the internal review board at the University of Florida. Acini were cultured in 3-D in Matrigel to allow dedifferentiation into ductal-like cells as previously described [46]. Briefly, acini passaged though a series of cell strainers (500, 300, 200 then 100 µm) was centrifuged at low speed and embedded into 125 µl Matrigel and cultured in complete media (50:50 DMEM:F12K containing 0.1 mg/ml soybean trypsin inhibitor (Sigma) and 10% FBS) per well of a plastic, 12-well ibidi chamber slide. The dedifferentiated phenotype (e.g., presence of ductal structures) was monitored microscopically for up to 5 days and then 500 nM TSA was added for 5 additional days.

### Compound treatments

For the ADM inhibition experiments, LLL12B and TSA (Sigma-Aldrich), were added to the p48^Cre/+^ organoids following 24 h of seeding and compound-containing fresh media was replenished every other day. Since the KC mouse organoids transdifferentiated faster than the p48^Cre/+^ mouse organoids, the KC mouse organoids were treated with LLL12B or TSA on the same day of seeding with no media replenishment. Microscopic adhesive grids (Sigma-Aldrich) were adhered to the bottom of the plate and the total numbers of ductal and acinar clusters were microscopically counted at ×4 magnification. These counts were performed after 4 days of culture of the p48^Cre/+^ organoids and after 2.5 days for the KC organoids. The percentage of ducts (% ADM) was calculated as the number of (ducts)/(number of acinar clusters + ducts) × 100. For the ADM reversal experiments in p48^Cre/+^ mouse organoids, ADM was allowed to occur over 3 days and then the cultures were exposed to increasing concentrations of LLL12B or TSA for 3 additional days of culture. ADM reversal in KC mice was performed by allowing the KC mice to undergo ADM for 2 days and then the cultures were exposed to increasing concentrations of LLL12B or TSA for 2 additional days.

### Cell viability

Cell viability after exposure to LLL12B or TSA was measured using the MTT assay (Sigma) following the manufacturer’s protocol. Organoid viability was also assessed using calcein AM (Thermo Fisher) by staining the organoids with a 10 µM solution in PBS for 0.5 to 1 h before imaging with epifluorescence microscopy.

### Exocrine cell 3-D morphology

Exocrine cell 3-D structure and amylase and Krt19 protein expression were determined following drug treatments using laser scanning confocal microscopy. Cells were mixed with Matrigel and media and plated in 12-well chamber slides (Ibidi). Following drug treatment, cultures were fixed in fresh 4% paraformaldehyde in PBS and immunostained for amylase or Krt19 as described in the supplemental methods section. For high content imaging, 20 µl of the ice-cold Matrigel cell mixture was pipetted into 384 well cell culture plates. After 30 min solidification of the Matrigel at 37 °C, 40 µl of media was added. Cultures were treated similar to those plated in 96-well plates, only 40 µl of media was used for the drug treatments. The cultures were imaged using high content imaging (Harmony 4.8, PerkinElmer) after ADM had occurred and after the TSA treatments. A total of 15 images were collected and stitched together using ImageJ software.

### RNA isolation and quantitative gene expression

As Matrigel contains degraded RNA, quantitative gene expression of the organoid cultures required first separation of the cells from the Matrigel using a published protocol as described [47]. Cellular pellets were lysed with TRIzol reagent (Thermo Scientific). Total RNA was isolated using the miRNeasy protocol (Qiagen). The integrity of the RNA was determined using the TapeStation RNA Screen Tape Analysis and routinely produced RNA Integrity Number (RIN) of 6 or higher. Sixty ng of total RNA was converted to cDNA in a 20 µl RT reaction using random primers and MMLV reverse transcriptase (Thermo). qPCR was performed using the QuantStudio™ 7 Flex Real-Time PCR System (Thermo). Data are presented using the 2^−ΔΔCT^ method relative to untreated control and normalized to 18S rRNA [48]. Primer sequences are provided in Supplemental Table 1.

### Immuno blotting

Western blotting for pStat3, Stat3, and α-Tubulin was performed using standard techniques. A detailed explanation of the methods may be found in the supplementary data section. An image of the full and uncropped western blot is provided in SFig. 1.

### Whole transcriptomic sequencing

Triplicate cultures of KC mouse acini underwent ADM for 2 days. Five hundred nM of TSA or LLL12B was added to the cultures for an additional 2 days. The transcriptomic contents of cells from the LLL12B, TSA, or day 4 untreated controls was determined. RNA sequencing libraries were synthesized as previously described [46] and sequenced using Illumina NovaSeq6000. An average of 61 million paired-end reads per sample were obtained after demultiplexing. Curated datasets were posted to the Gene Expression Omnibus (GEO) repository under accession number GSE195560. Reads were quality trimmed at Q20 (sequencing quality score of 20) and filtered to remove sequencing adapters using Trimmomatic (v.0.39). Trimmed and filtered reads were then aligned to the mouse reference genome (GRCm39) with its corresponding annotation file obtained from the European Bioinformatics Institute (EMBL-EBI). Alignment and quantification were done using STAR (v.2.7.9a). Gene counts were then imported to edgeR (v.3.26, R v.4.1.2) for normalization, principal component analysis (PCA), and differential expression analysis. We considered a transcript differentially expressed if its edgeR FDR adjusted *P*-value <0.05.

### Data analysis

Concentration-response curves were generated in GraphPad Prism 8.2.1 software, using nonlinear regression analysis for % ADM or % viability, normalized to the untreated control. The data were fit using the inhibitor-dose response model with variable slope and the EC_50_ and IC_50_ values were calculated. The results are presented as mean ± SD and were analyzed using an unpaired Student’s *t*-test. RNA sequencing data were analyzed as previously reported [46]. Pathway analysis was performed using Ingenuity Pathway Analysis (Qiagen) on ~4000 differentially expressed genes (fold change greater or less than 1.5-fold and FDR *p* value <0.05).

## Supplementary information

Supplemental Material

Supplemental Table 3

## Data Availability

The datasets generated during and/or analyzed during the current study were posted to the Gene Expression Omnibus (GEO) repository under accession number GSE195560. GEO Accession viewer (nih.gov).
