# Supplementary material for: Pharmacological inhibition and reversal of pancreatic acinar ductal metaplasia
Source: Cell Death Discov. 2022 Sep 2;8:378. doi: 10.1038/s41420-022-01165-4 (PMC9440259; doi:10.1038/s41420-022-01165-4)
Supplement: Supplementary file 1 — Supplemental Material [file 41420_2022_1165_MOESM1_ESM.docx]

**SUPPLEMENTAL METHODS AND DATA**

**For**

**PHARMACOLOGICAL INHIBITION AND REVERSAL OF PANCREATIC ACINAR DUCTAL METAPLASIA**

**Lais da Silva^1,*^, Jinmai Jiang^1,*^, Corey Perkins^1,*^, Kalina Rosenova Atanasova^2,3^, Julie K. Bray^4^, Gamze Bulut^1^, Ana Azevedo-Pouly^5^, Martha Campbell-Thompson^4^, Xiaozhi Yang^2^, Hesamedin Hakimjavadi^4^, Srikar Chamala^4^ _,_ Ranjala Ratnayake^2.3^, Raad Z. Gharaibeh^6,7^, Chenglong Li^2,3^, Hendrik Luesch^2,3^ and Thomas D. Schmittgen^1,†^**

**^1^Department of Pharmaceutics, College of Pharmacy; ^2^Department of Medicinal Chemistry, College of Pharmacy; ^3^Center for Natural Products, Drug Discovery and Development; ^4^Department of Pathology, Immunology, and Laboratory Medicine, College of Medicine; ^5^Department of Surgery, University of Arkansas for Medical Sciences; ^6^Department of Medicine; ^7^Department of Molecular Genetics and Microbiology, University of Florida, Gainesville, FL, USA**

**Exocrine cell 3-D morphology**

Exocrine 3-D structure and amylase and Krt19 protein expression were determined following drug treatments using laser scanning confocal microscopy. Cells were mixed with Matrigel and media and plated in 12-well chamber slides (Ibidi). Following drug treatment, cultures were fixed in fresh 4% paraformaldehyde in PBS (Sigma) for 30 minutes at room temperature followed by three washes for 15 minutes each with 1xPBS. Slides were stored at 4° C until immunostaining. Immunostaining was performed using modified iDISCO method (Visikol) with steps performed at room temperature on a rocker for ≤ 15 minutes each step. Organoids were dehydrated through ethanol (50%, 80%, 100%) followed by 80% ethanol/20% DMSO and rehydration to PBS buffer containing 0.2% Triton-X 100 (PBST). Organoids were incubated in penetration buffer followed by blocking buffer then in primary antibodies (Mouse anti-KRT19 (1:100, DAKO, M088801-2); Rabbit anti-α-Amylase (1:100, Sigma, A8273-1VL)) in antibody diluent for two hours. Following 5 washes in PBST, secondary antibodies (Donkey anti-mouse AF555, Donkey anti-rabbit AF488, 1:500 each (Thermofisher)) were applied in antibody diluent and slides were incubated in the dark on the rocker for 1 hour at room temperature. Slides were incubated with Hoechst S769121 (1:500 in PBST, Abcam ab138903) for 15 minutes followed by 4 PBST washes. Slides were mounted with HISTO-M mountant (Visikol) following chamber removal and dried overnight. Microscopy was performed with a Zeiss 710 using 40x objectives and 405, 488, and 562 lasers with filters for Hoechst, AF488, and AF555 fluorophores, respectively. Single plane images and z-stacks (10-30 µm, 1-2 µm slices) were obtained and maximum intensity projections of the x-stacks.

**Immuno blotting**

KC ADM cultures (two wells of a 48 well plate per treatment) were treated with 1 µM LLL12B for 6 hrs. Following treatment, the Matrigel and cells from the replicate cultures were combined and the Matrigel was separated from the cells as described (Da Silva L, et al., MethodsX 2020, 7: 100966). Cells were lysed in Bolt LDS Sample Buffer (Novex) and Bolt Sample Reducing Buffer (Novex) then heat denatured at 70˚ C for 10 min. Protein concentration was quantified using the BCA Assay (Thermo). Protein (20 µg) was resolved on a 4%-12% Bis-Tris Plus Gel (Invitrogen) then transferred to a PDVF membrane using a Trans-Blot Turbo Transfer System (BioRad). The membrane was incubated with primary antibody overnight at 4˚ C. Membranes were incubated in goat anti-rabbit-HRP antibody for one hour at room temperature and imaged using the Amersham ECL Detection system (GE Healthcare). The following primary antibodies were used: Rabbit anti-phospho-Stat3 (Tyr705) mAb (1:500, Cell Signaling 9145T); rabbit anti-Stat3 mAb (1:1000, Cell Signaling, D3Z2G); rabbit α Tubulin (3:2000, Cell Signaling, 2144S). An image of the full and uncropped western blot is provided in SFig. 1.

**Supplemental Figure 1**

**Supplemental Figure 1. LLL12B inhibits pStat3 phosphorylation.** Western blot of KC mouse organoids from untreated (UnTx) or 1 µM LLL12B treated (Tx) for 6 h. After 2 hours, the untreated and LLL12B-treated cells were stimulated by IL-6 (25 ng/ml) in an attempt to stimulate pStat3. The cells were harvested at 30 minutes and analyzed by Western blot (20 µg protein loaded per lane). The phosphorylation site for pStat3 is Tyr705. Shown (upper) is the full length blot that was cut into fourths and immunoblotted against Stat3, pStat3, Tubulin or GAPDH (the blot was exposed for 1 min). Shown below is the same blot that was exposed for 30 mins to enhance the image of the tubulin. Dashed boxes represent the portions of the blots that are presented in Fig. 1G.

**Supplemental Figure 2**


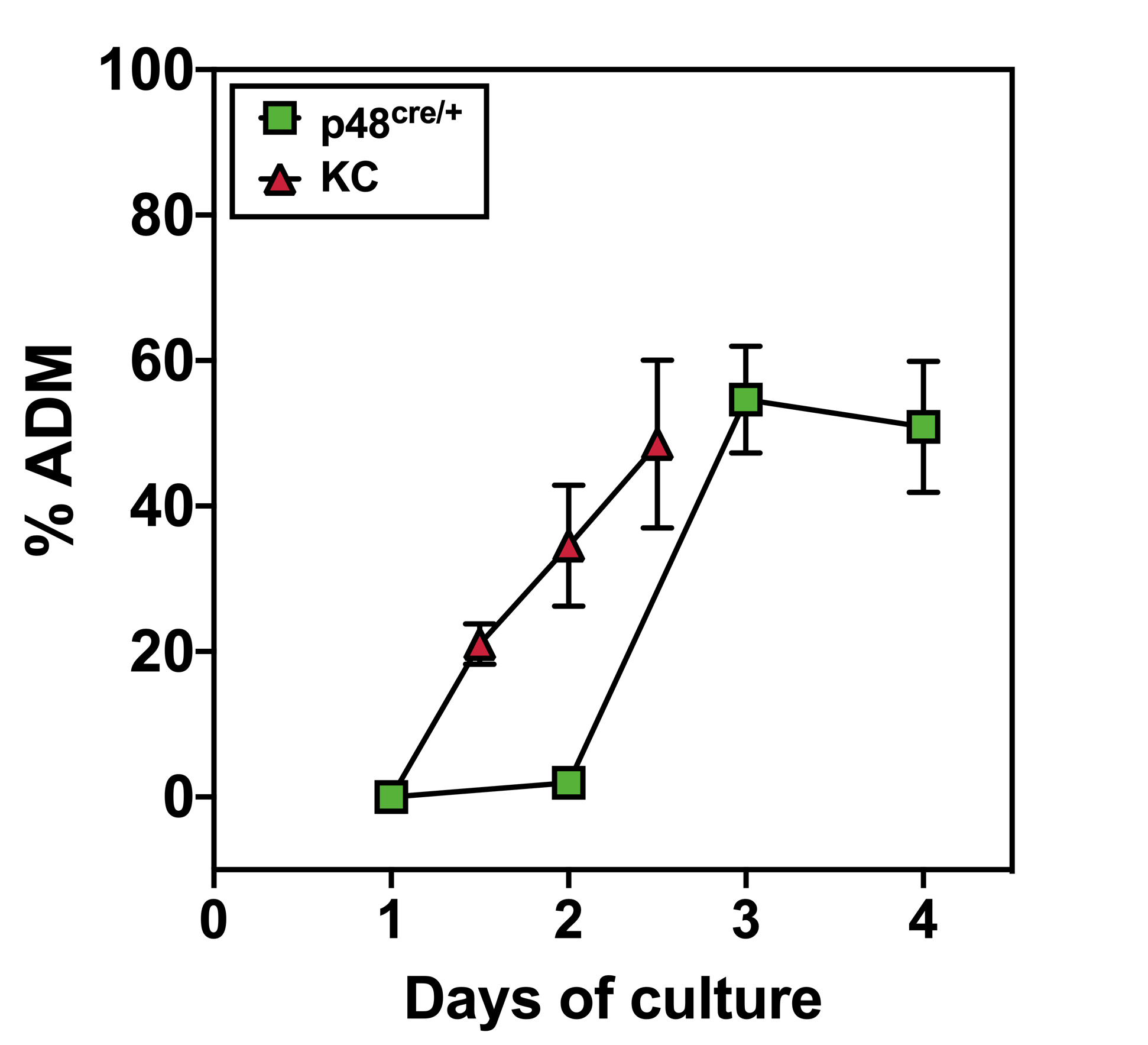


**Supplemental Figure 2. Duct formation versus time of culture in p48^Cre/+^ and KC mouse organoids.** Pancreatic acini from p48^Cre/+^ and KC mice were cultured as organoids. The number of ducts and acinar clusters were microscopically quantified over the culture period and the % ADM was calculated as described in Methods.

**Supplemental Figure 3**

**Supplemental Figure 3. Reversal of ADM by TSA in KC mouse acini.** KC mouse pancreatic acini underwent ADM over 2 days of culture (A) and were then exposed to DMSO (B) or 10 µM TSA (C) for 3 additional days followed by Calcein AM viability staining. Shown are the high content images, with the enlarged sections (dashed lines) in the lower panels. Scale bars in the top images represent 500 µm. Expression of acinar (D) and ductal (E) genes from TSA treated KC mouse pancreatic organoids following the identical treatment as in (A-C). Data are presented as fold-change normalized to 18S rRNA and relative to the untreated control. Mean ± SD from duplicate experiments.

**Supplemental Figure 4**

**Supplemental Figure 4. Acinar and ductal changes during re-differentiation of KC mouse organoids by TSA.** KC mouse pancreatic organoids were plated onto Matrigel and were fixed following one day of culture (Control day 1). After two days of culture the organoids were either untreated (Control day 4) or exposed to 500 nM TSA (TSA Day 4) and cultured for 2 additional days before fixation and immunostaining. Shown are the individual channels and the composite images. Scale bars: 50 µm.

**Supplemental Figure 5**
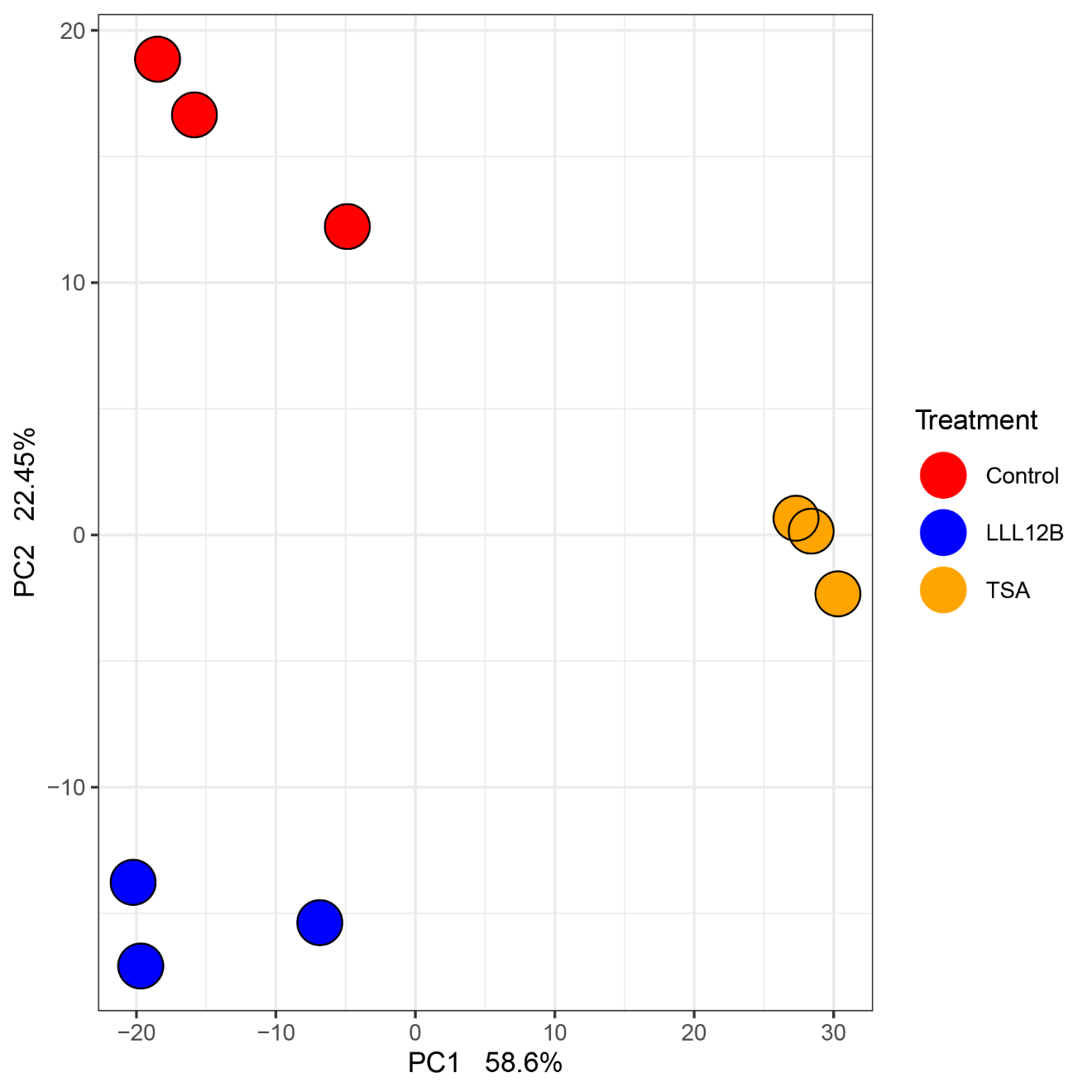


**Supplemental Figure 5. Principle component analysis from control, TSA and LLL12B ADM reversal treatments of KC mouse acini.** KC mouse pancreatic acini underwent ADM over 2 days of culture and were then exposed to 500 nM of LLL12B for 2 additional days. RNA isolated from the treated and day 4 untreated control were subjected to Illumina NovaSeq6000 whole transcriptome sequencing. Shown is the PCA plot of the resulting data.

**Supplemental Figure 6**


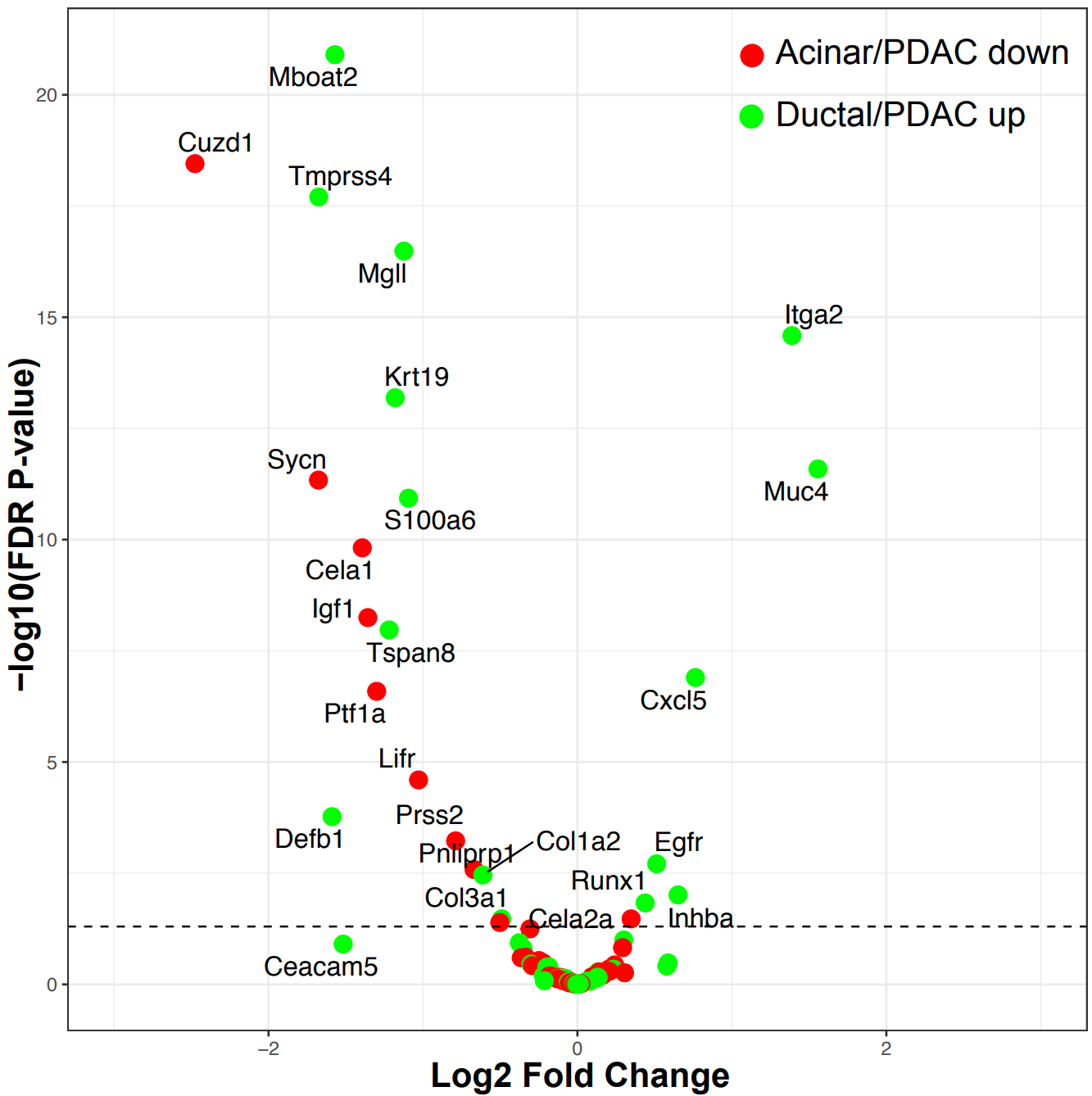


**Supplemental Figure 6. Volcano plot of differentially expressed genes from LLL12B-induced ADM reversal of KC mouse acini.** KC mouse pancreatic acini underwent ADM over 2 days of culture and were then exposed to 500 nM of LLL12B for 2 additional days. RNA isolated from the treated and day 4 untreated control were subjected to Illumina NovaSeq6000 whole transcriptome sequencing. Shown are the expression of a selected set of 82 genes which are the mouse equivalents to those previously identified as associated with the pancreatic acinar/ductal phenotype or genes associated with the onset or progression of PDAC as described in the Methods section.

**Supplemental Figure 7**


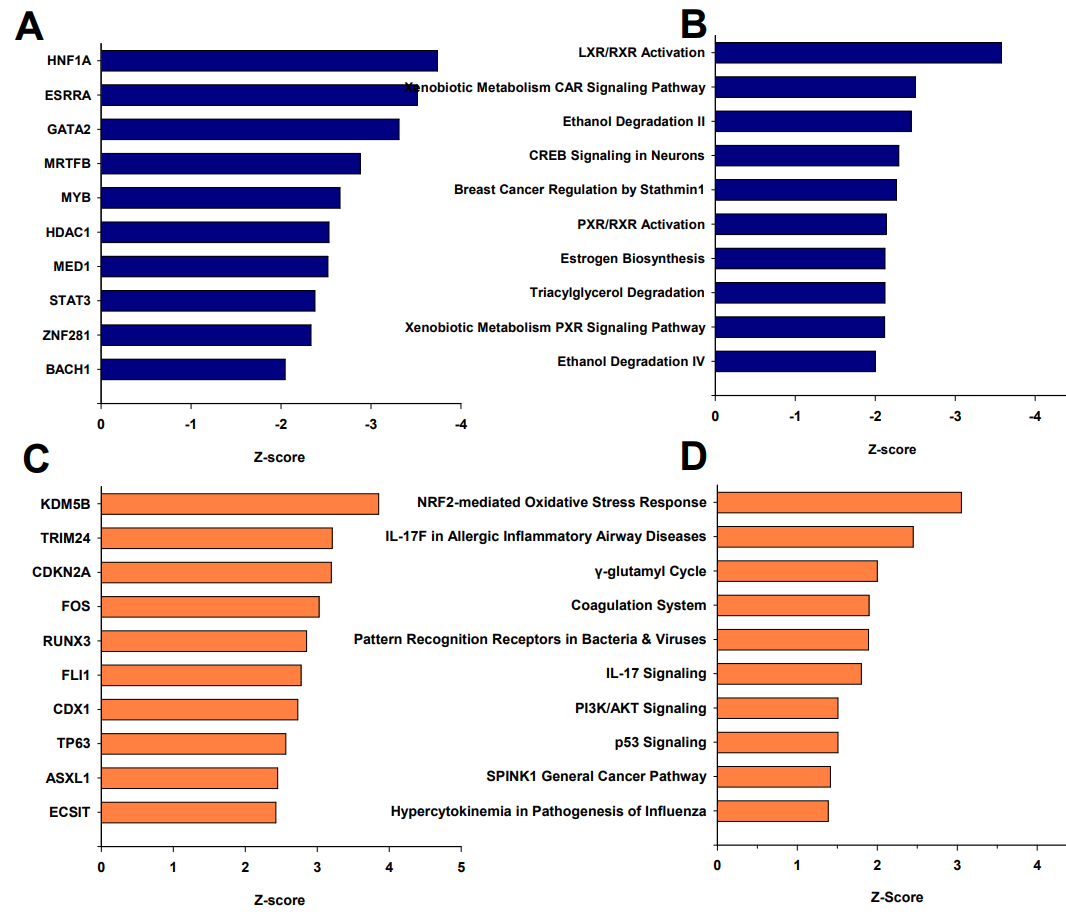


**Supplemental Figure 7. Pathway and upstream analysis during the reversal of ADM by LLL12B in KC acinar cells.** RNA sequencing data from the ADM reversal in KC mouse acini following 500 nM LLL12B treatment was analyzed using Ingenuity Pathway Analysis. (A,C) Top 10 transcription factors involved in the upstream regulation ranked by Z-score. (B,D) Most highly ranked signaling pathways ranked by Z-score. Blue, inhibited and orange, activated pathways.

| **Supplemental Table 1** | | |
| --- | --- | --- |
| **Gene** | **Sense Primer Sequence** | **Antisense Primer Sequence** |
| Amy2a | TTGCCAAGGAATGTGAGCGAT | CCAAGGTCTTGATGGGTTATGAA |
| Cpa2 | GATCAAGAGCGTGAAGAGATGC | AGCCACGAGGTTATCCATTTCT |
| Cela1 | AGTACCAGTATGGAGGATCATGG | CAACCACTCGATAAGTCATGGG |
| Krt19 | GGGGGTTCAGTACGCATTGG | GAGGACGAGGTCACGAAGC |
| Krt7 | AGGAGATCAACCGACGCAC | CACCTTGTTCGTGTAGGCG |
| Sox9 | CTGCACAACGCGGAGCTC | ACGAAGGGTCTCTTCTCGCTCT |
| 18S rRNA | GTAACCCGTTGAACCCCATT | CCATCCAATCGGTAGTAGCG |
